# The resonance phenomenon in population persistence: can the same theory guide both national security policies and personalized medicine?

**DOI:** 10.3325/cmj.2014.55.93

**Published:** 2014-04

**Authors:** Zvia Agur

**Affiliations:** Institute for Medical BioMathemetics (IMBM), Bene Ataroth, Israel

## Abstract

The theory of resonance in population persistence proposes that the survival of a population that is exposed to externally inflicted loss processes (disturbances) during part of its life cycle is dependent on the relation between the average period of the disturbances and the average generation time of the population. This suggests that the size of a population can be controlled by manipulating the period between external disturbances. This theory, first formalized in a study of intertidal Red Sea mollusks exposed to periodic storms, has been found to apply to such seemingly disparate phenomena as the spread of a pathogen among susceptible individuals and the response of malignant cancer cells to chemotherapy. The current article provides a brief review of the evolution of the resonance theory into a tool that can be applied to designing vaccination policies – specifically, in preparedness for bio-terrorism attacks – and in personalized medicine. A personalized protocol based on the resonance theory was applied to a cancer patient, stabilizing his tumor progression, relieving his hematopoietic toxicity, and extending his survival.

A few short decades ago, mathematics and biology were considered to be entirely disparate fields: biology was a “wet science” with little use for theory. Yet nowadays it is difficult to imagine the field of biology without mathematical modeling. Models enable researchers to simulate the progression of cancer and other diseases on a cellular level, and to predict the response of patients to familiar and prospective treatment regimens (1). In epidemiology, mathematical models are used to predict how new pathogens will spread in populations of unvaccinated individuals (2). Clearly, a mathematical model can provide practical insights into the specific phenomena it addresses –indicating, for example, how frequently a drug should be administered, or the number of people who are expected to contract a new disease. But perhaps the most successful models are those that provide a deeper understanding of the phenomena at hand, enabling researchers to draw fundamental conclusions regarding the nature of biological systems.

This article discusses how mathematical modeling revealed a single physical process – the resonance phenomenon in population persistence – underlying a large number of biological processes that seemed entirely unrelated to one another, aside from the fact that they were subject to the influence of external forces. Agur and Deneubourg (3) first discovered this phenomenon when they developed a mathematical model to investigate how mollusk populations in the Red Sea are able to persist despite periodically being decimated by severe storms. Agur and Webb later expanded the model into a general theory (4-6) and, further, asserted that the resonance phenomenon characterized the spread of pathogens in a human population under vaccination, as well as the progression of various types of cancer under therapy. As will be shown below here, this insight enabled Agur et al to suggest definitive strategies for influencing these biological processes. In particular, Agur et al discussed how policy makers can devise resource-efficient vaccination strategies in the face of epidemics and even bioterrorism threats (7-9). Additional studies, relying on the same theory, have theoretically and experimentally shown that a chemotherapeutic dosing schedule can be planned such that drug efficacy is maximized and toxicity is minimized (10,11). These studies indicated that the optimal dosing schedule for a given patient is dependent on specific characteristics of the patient's internal biological processes, suggesting that drug regimens ought to be determined for each patient on an individual level. This notion, which has since received substantial theoretical support, was tested empirically both in cellular and murine experiments and in the clinic (10-12). Drawing from resonance theory, Gorelik et al (12) designed a personalized dosing regimen for a cancer patient whose treatment had previously been unsuccessful, and the new schedule substantially improved the patient's clinical outcomes. The current article provides a brief review of the evolution of the resonance theory into a tool which can be used in determining vaccination policies – and, specifically, in preparedness for bio-terrorism attacks – and in personalized medicine.

## The theory of resonance in population persistence

When a system is oscillating at a given frequency, even small periodic driving forces – if applied at the correct frequency – can yield large amplitudes of oscillation. This is called *resonance*. To understand how this works, imagine a mother pushing her child on a swing: if she pushes the swing only once, the swing will continue to go back and forth at a natural frequency (or period). If she pushes the swing every time it goes back, at the peak of its trajectory, it will continue to go back and forth at the same natural frequency, but the amplitude of the swing will increase from push to push. Notably, this will occur even if the mother gets tired and only pushes the swing every second time it goes back, or every third time – that is, the amplitude of the swing will continue to increase if the period of her push divided by the period of the swing is a whole number. But if she pushes the swing at times that are completely different from the period of the swing – eg, at random (such that the period of the push divided by the period of the swing is an irrational number) – the amplitude of the swing will slowly go down to a minimum.

Most biological systems can be characterized as “oscillating,” that is, they entail cyclicality. Individuals are born into a population, reproduce, and die. This is true for mollusks, healthy somatic cells, and cancer cells alike. The process by which an epidemic spreads in a population can also be characterized as oscillatory: a pathogen is introduced into a population, people contract the disease and spread it to others, and the number of infected individuals rises to a maximum, and then gradually diminishes as people acquire immunity (or die). Yet a few years down the line, if no additional action is taken, the disease resurfaces, as new non-immune people enter the population (2). The oscillating nature of biological systems suggests that we can exploit the phenomenon of resonance to manipulate their dynamics. That is, by applying force at opportune moments, it is possible to enhance the growth of desirable populations (such as healthy tissue cells), or to eliminate undesirable populations (such as cancer cells).

**Figure 1:**
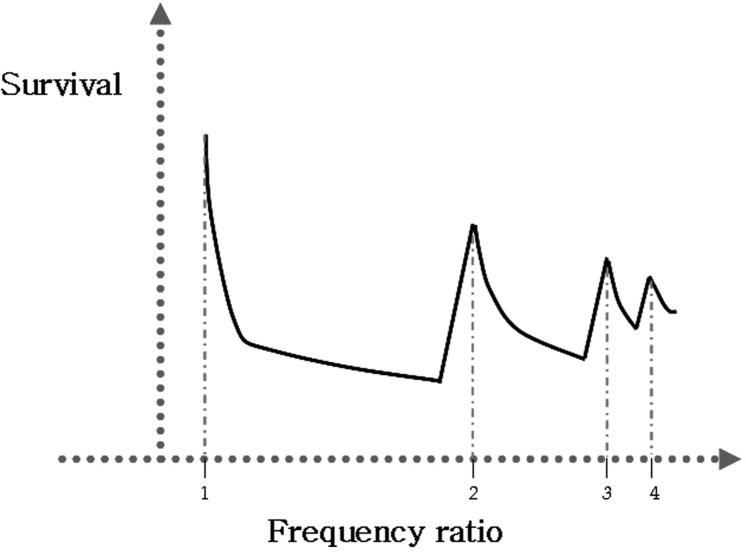
A schematic illustration of the resonance phenomenon. Survival of a population is plotted as a function of the ratio between the inherent population periodicity and the disturbance periodicity, τ/δ+ω, where τ is the generation time of the population and δ+ω stands for the average period of the disturbance process.

Agur (4) and Agur and Deneubourg (3) were the first to mathematically model and prove the existence of the resonance phenomenon in population persistence. Their models aimed to shed light on the means by which populations are able to survive despite being periodically exposed to environmentally inflicted loss processes, or “catastrophes.” Agur and Deneubourg (3) focused on the case of intertidal Red Sea mollusks, which are periodically exposed to harsh storms. These storms obliterate the adults, which are sessile on the rocks, but they do not harm the plankton-like juveniles, which drift freely in the sea. The authors investigated the relationship between the durations of such catastrophes and the duration of the resistant, juvenile stage of the mollusk life cycle. Analysis of their model showed that the population was most vulnerable to randomly occurring catastrophes (ie, the population's time to extinction is minimized) if the ratio between the average generation time in the population and the average period of the catastrophe is irrational (Figure 1). Agur (4) extended this model into a general theory, asserting that the survival of any population that is susceptible to imposed loss processes during part of its life cycle is dependent on the relation between the average period of the disturbances and the duration of the life cycle of the population. Resonance – that is, maximum population growth – occurs when the average period of the imposed loss process is an integer or fractional multiple of the inherent period of the population. This suggests that the size of a population can be controlled by manipulating the duration of the period between external disturbances. More generally, a dynamic process that oscillates in the number of individuals who are susceptible to a given disturbance can be efficiently controlled by imposing recurring disturbances of a predetermined period.

## Using the resonance phenomenon to identify optimal vaccination policies

### Pulse vaccination policy

A key objective of epidemiology, the study of the patterns of disease spread in populations, is to develop optimal vaccination policies against infectious diseases. In this domain, acknowledgment of the resonance phenomenon in population persistence can have far-reaching implications.

The mathematical SEIR (Susceptibles, Exposed, Infectious, Resistant) model is a straightforward means of describing the spread of an infectious disease in a population (2). This model divides the population into four compartments: (i) Susceptible individuals (S), who have not yet been infected and are vulnerable to the disease; (ii) exposed individuals (E), who have been infected but cannot yet infect others; (iii) infectious individuals (I), who can infect others; and (iv) resistant individuals (R), who cannot be infected by the disease. Individuals can become resistant – ie, immune – either by contracting and overcoming the disease, or by being vaccinated.

**Figure 2:**
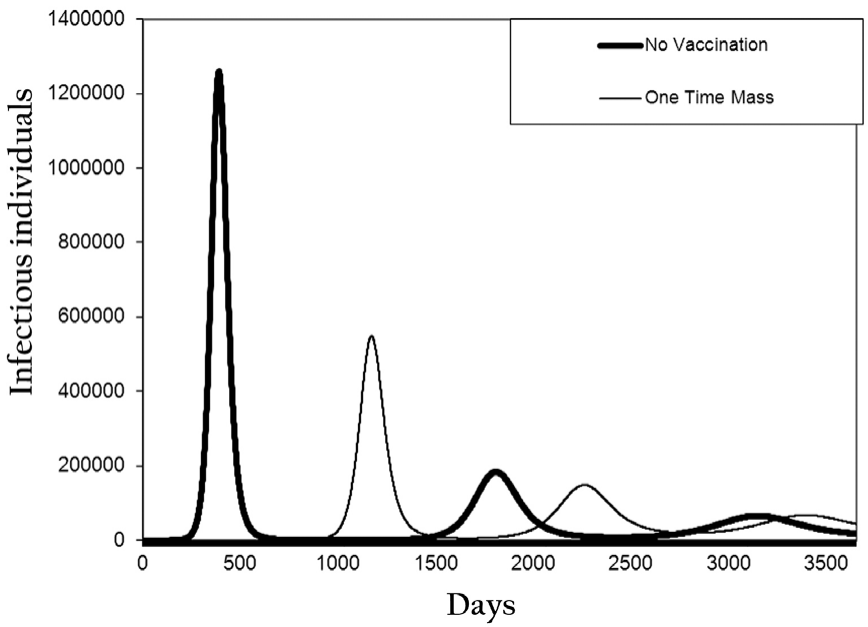
Simulation results of an adapted SEIR model (8): number of infected individuals over time when smallpox is introduced into the Israeli population by one infective carrier. Thick line: natural disease dynamics (no vaccination strategy is implemented). Thin line: disease dynamics after implementation of one-time mass vaccination strategy (population is vaccinated at maximum capacity for three days following the attack).

When applied to a large unvaccinated population over a long period of time, the SEIR model describes a pattern of recurring epidemics of diminishing scope (Figure 2). In a single epidemic, the number of susceptible individuals increases quickly as people are exposed to the disease and then decreases through the natural process of infection and acquisition of natural immunity, until the number of susceptible people becomes so small that it practically prevents further contact between susceptible and infectious persons, thus leading to the termination of the current epidemic. However, as new people enter the population (ie, are born into the population or enter through immigration), the number of susceptible people grows once again, and when its density is sufficiently high, a new introduction of the pathogen will cause a new epidemic to flare up. In this way, epidemics of infectious diseases become an oscillatory process. The actual trajectory of a disease in a given population – that is, how frequently it recurs and the number of individuals who will be infected in each outbreak – is dependent on the characteristics of the disease (eg, its virulence, the duration of time for which it is contagious) as well as the characteristics of the population (eg, diseases might be more likely to spread in large, crowded cities than in rural areas where people have little exposure to one another). These qualities are all captured in a single model parameter called the basic reproduction rate of the epidemic (2).

According to the resonance theory outlined above, it seems that the periodic dynamics of disease can be efficiently combated by a periodic vaccination policy (to be denoted pulse vaccination policy). In this case, the population being affected by the loss process is the population of susceptible individuals: this population is “diminished” by vaccination, because vaccinated individuals cease to be susceptible and become resistant.

In 1993, Agur et al developed a mathematical model with the goal of proposing a policy that would eradicate measles in Israel (9). The vaccination policy in place at the time was a typical cohort-based vaccination policy, ie, children were vaccinated once at the age of 15 months and again at the age of 6 years. However, this policy was insufficient to prevent measles from recurring every five years or so. Agur et al showed that vaccination of 1-7-year-olds every five years would prevent epidemics from recurring. Shulgin et al analyzed the model and showed that the pulse vaccination policy can lead to eradication of the epidemic, also withstanding seasonal variation (13). A strategy based on proposition by Agur et al was successfully implemented by the Israeli Ministry of Health.

An interesting observation that emerges from the study by Agur et al is that, intuitively, it seems as if the entire population should have been “covered” by the cohort-based vaccination policy that was in place. Yet herd immunity in the Israeli population was insufficient to prevent disease outbreak. This emphasizes that reality – in which many individuals are not vaccinated as a result of noncompliance, contraindications, or other factors – might not always correspond to an intuitive understanding of which vaccination strategies should be effective. The mathematical model by Agur et al captured the recurrence of epidemics and showed that periodic vaccination of a small portion of the population was a more effective means of ensuring that the disease does not recur in the population.

### Pulse vaccination in bioterrorism preparedness policies

The need to identify effective vaccination strategies became particularly salient in the early 2000s when, following the events of 9/11, bioterrorism suddenly became a serious concern. The US Centers for Disease Control and Prevention (CDC) was particularly concerned about the possibility of an outbreak of smallpox, owing to the ease of transmission of this agent, and the susceptibility to it in the previously unexposed population. A vaccine against smallpox (vaccinia) existed but was associated with significant adverse side effects (14). Furthermore, even if the population were to be vaccinated, little was known about how a smallpox bioterrorism attack would play out in a contemporary population. In particular, it was not clear what the disease’s actual basic reproduction rate would be, how the population would respond to an outbreak, how willing people would be to comply with vaccination campaigns, and how much vaccine would be available.

Two strategies being considered by policy makers at the time, included preliminary vaccination (ie, vaccinating the population before an attack had actually happened) and post-attack mass vaccination – that is, vaccinating the entire population immediately after disease outbreak. Clearly, neither approach is ideal – preliminary vaccination risks “wasting” vast resources if no attack actually occurs. Post-attack mass vaccination – perhaps the most intuitive response to such an event – is likely to be impeded by mass hysteria and vaccine stockpile shortage. At that period, several mathematical models were developed in order to analyze the problem of a potential smallpox outbreak in the human population and identify an effective vaccination policy (14-17). However, some models contradicted the recommendations of others, owing to a fundamental lack of knowledge regarding the characteristics of the virus. More worryingly, these models focused on preventing a single epidemic but did not evaluate what would happen several years down the line. Agur et al (8) showed, using the SEIR model, that preliminary vaccination and one-time mass vaccination strategies would not actually prevent epidemics from recurring several years following an initial attack. This might seem counterintuitive, given that these vaccination policies claim to “cover everyone” – yet, as discussed in the previous subsection, and as shown through the dynamics of the SEIR model (Figure 2), reality does not always correspond to intuition.

**Figure 3:**
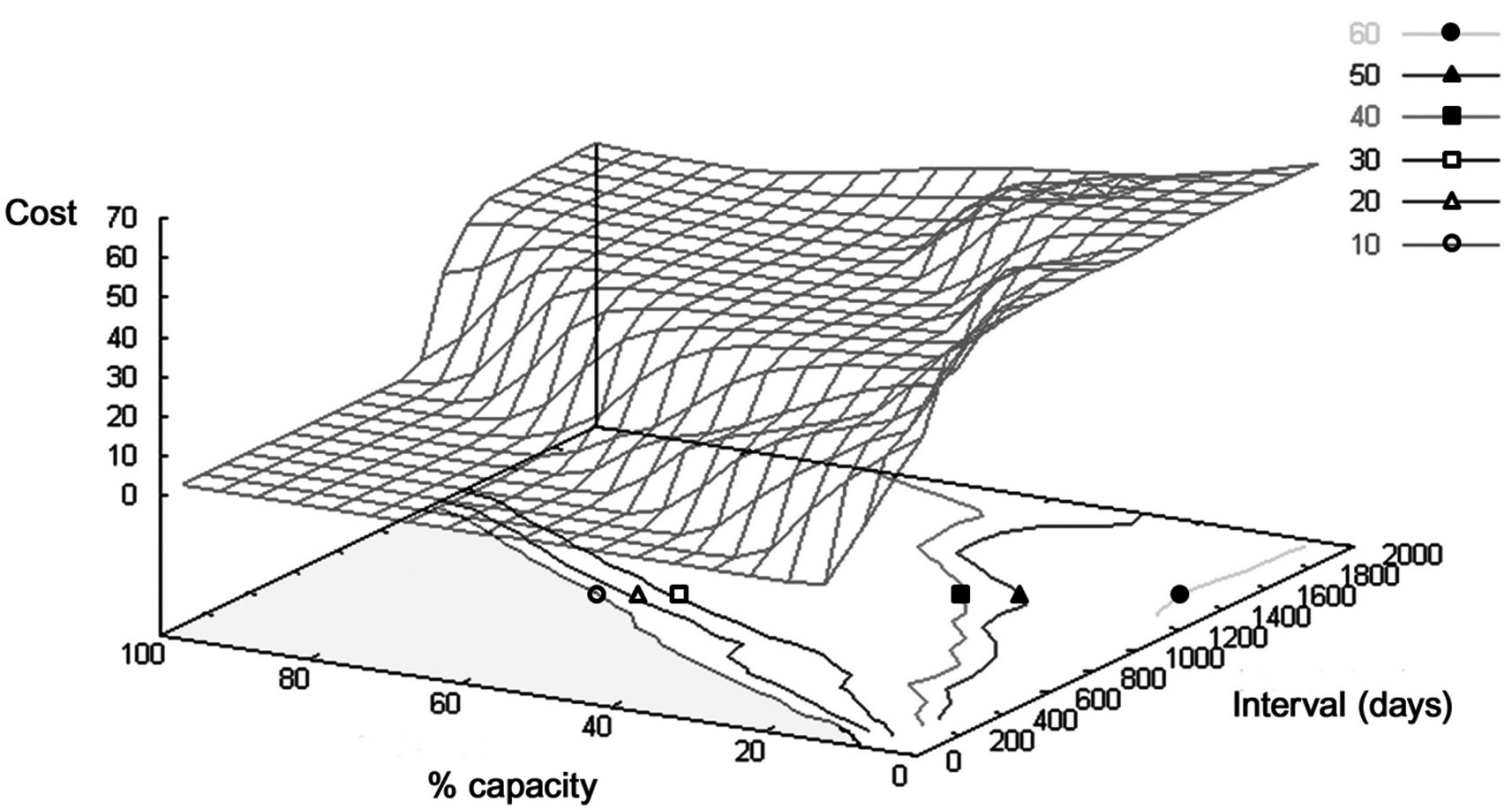
Simulated outcomes of a pulse vaccination strategy implemented in Agur et al (8): cost (calculated according to the number of infected individuals, in addition to other factors) as a function of percent vaccination capacity utilized and interval between pulses (in days), assuming that the disease's basic reproduction rate is 3. There is a critical ratio (diagonal contour) between capacity utilized and the interval, above which the strategy will be effective (shaded area) and below which costs rise suddenly.

Agur et al proposed a pulse vaccination policy, wherein once every few months or years following disease outbreak, a certain percentage of the population is vaccinated. Under this policy, the actual details of the vaccination strategy – namely, the percentage of the population vaccinated and the period between vaccination campaigns – are determined after the smallpox pathogen has been introduced into the population, when more information on the behavior of the disease is available. They showed that a pulse strategy would not only be more effective over the long term compared with preliminary or one-time mass vaccination but would also be more practical: the success of the strategy is not dependent on full and immediate compliance and vaccine availability. Furthermore, the strategy offers flexibility, which is crucial in situations characterized by such high uncertainty. Specifically, there exists a critical ratio between the percentage of the population vaccinated per day and the inter-pulse interval, above which the pulse vaccination strategy will suffice to prevent subsequent epidemics. As long as this ratio is maintained, the actual period and the number of people vaccinated can be adjusted according to actual compliance or vaccine availability; for example, if fewer people than desired are vaccinated during an individual campaign, it is possible to compensate by shortening the time period until the next campaign (Figure 3).

## Using the resonance phenomenon to identify personalized chemotherapy regimens

### Resonance in chemotherapy

Cancer is treated primarily by cell-cycle phase-specific chemotherapeutic drugs. These drugs destroy cells that are in a specific proliferative phase of the cell-cycle, whereas cells in other cycle phases, quiescent or non-proliferative cells, are resistant to treatment. After treatment is administered, some unharmed cells become proliferative, and the drug-susceptible cell population is replenished. Clearly, then, like populations of inter-tidal organisms, or populations of individuals who are susceptible to an infectious disease, drug-susceptible cancer cell populations are characterized by periodic dynamics. Thus, according to the resonance theory, these populations are likely to be efficiently controlled through control of a periodic loss process inflicted by chemotherapeutic drugs. Likewise, when considering cancer treatment, one should also consider the healthy host cells, which are also adversely affected by treatment (in a cell-cycle-phase-specific manner). The resonance theory suggests that it is possible to choose a dosing schedule – that is, a frequency of catastrophes – that will eliminate the population of malignant cells while maintaining the population of healthy cells at a maximum. Specifically, the dosing period should be a fractional multiple of the average cell-cycle duration of healthy host cells but not of the cell-cycle duration of cancer cells (10,18). This approach is a departure from the well-known “dose-dense” paradigm proposed by Norton and Simon (19). Norton and Simon argued that the efficacy of cancer therapy can be enhanced by increasing the dosing rate, for example, by applying the maximum tolerated dose and decreasing the inter-dosing intervals. This paradigm is based on the assumption that cancer growth obeys the Gompertz law (where population growth is described by a sigmoid function, growth being slowest at the start and end of a period), and that the rate of tumor regression induced by chemotherapy is proportional to the growth rate of an untreated tumor of the same size (18,19). Although this paradigm has been used to treat cancer effectively (20), Norton and Simon never validated the Gompertz model, that is, they never checked whether in real life tumor growth actually obeys the sigmoid function comprising the Gompertz law, so that the dose-dense paradigm can be generalized.

The validity of the resonance-based approach has been supported both theoretically and empirically. Theoretical studies include, among others, those of Agur et al (6,10) and Dibrov et al (21), which modeled treatment of cancer with cell-cycle phase-specific drugs. These studies used highly simplified models of a cancer patient, assuming that tumor cells and target host tissues differed only in terms of their cell cycle parameters (all studies ignored the spatial arrangement of these cells). This approach reduced the complex physiological and pathological dynamics of tumor progression under different chemotherapy regimens into one essential variable: the ratio between the rate of tumor growth and the growth rate of healthy tissues.

Arnon and Schechter (10) considered an abstract case in which drug efficacy was stepwise “infinite,” ie, all cells in the vulnerable cycle phases were instantly eliminated during the fixed period in which the drug was effective, while no cells were affected during the drug-free interval. This model, which assumed stochastic inter-dosing intervals, showed a clear advantage for population growth when the period of the loss process coincided with an inherent population reproduction period. Cojocaru and Agur (6), who also relied on a probabilistic dosing interval, considered a more realistic case, in which drug efficacy was “finite” and driven by drug decay kinetics. Analysis of this model provided explicit formulas for the growth or decay of the tumor cell population, which further supported the existence of the resonance phenomenon. Kheifetz et al (22) proved that the superior efficacy endowed by applying fully periodic drug regimens according to the Resonance Theory was steadily maintained over time.

Dibrov et al (21) relied on systems of deterministic differential equations (rather than on a stochastic approach) and showed that when the mean cell-cycle times of normal and tumor cells differ considerably, optimal drug schedules can significantly improve therapeutic efficacy, when drug periodicity is close to the mean cell-cycle time of the normal population and is practically independent of the tumor cell-cycle parameters. In this case, identification of the optimal treatment regimen heavily depends on precise estimation of the mean cell cycle duration of both cancer and host cells.

In vitro and in vivo experiments have provided further support to the proposition that drug timing can be manipulated to minimize toxicity and maximize drug efficacy. In vitro experiments (10), in which cells were exposed to short pulses of cytosine arabinoside (ara-C) – an S-phase-specific cancer drug – tested the dependence of cell population growth on the inter-dosing interval. Experimental results showed that, regardless of the total drug dose or the experimental procedures, drug schedules whose periodicity was an integer multiple of the average population cell-cycle duration were associated with significantly higher population growth rates, as evaluated by rates of DNA synthesis.

Ubezio et al (11) investigated whether a resonance-based dosing approach could be feasible using the clinical methods available at the time. They administered short pulses of ara-C, with different dosing schedules, to lymphoma-bearing mice. The schedules varied in terms of the distributions of the inter-dosing intervals, and included stochastically-determined schedules; the total dose and total treatment duration were kept constant across treatment conditions. Toxicity was evaluated by spleen weight, peripheral blood measurements, and the proportion of bone-marrow cells in the S-phase gate of the DNA content distributions. The observed response of mice to the administered treatment schedules showed that a schedule in which the duration of the inter-dosing intervals was random inflicted higher cytotoxicity compared with a regimen of constant inter-dosing intervals, as reflected in bone marrow cells (see Figure 7 in [1]) and in mice survival (see Figure 9 in[1]). The experimental results suggest that myelotoxicity is minimized when the inter-dosing interval is a multiple of the inter-mitotic time of bone-marrow stem and progenitor cells, thereby supporting model predictions.

In another study, Skomorovski and Agur (23) investigated the resonance phenomenon from another angle – appropriate timing of dosing schedules aimed at boosting the growth of healthy hematopoietic host cells rather than eliminating cancerous cells. The study focused on the hormone thrombopoietin (TPO), also known as megakaryocyte growth and development factor. This agent can potentially be used to attenuate thrombocytopenia but is associated with severe immunogenicity, which limits its therapeutic use. The authors developed a mathematical model and a simulation tool that showed that platelet counts similar to those obtained with standard, immunogenic TPO dose scheduling can also be obtained with safer periodic schedules of significantly reduced doses that are appropriately timed. Skomorovski et al (24) later validated this prediction in mouse and monkey experiments. Specifically, in a two-arm mouse experiment, they showed that platelet profiles obtained with a single intraperitoneal dosing of recombinant mouse TPO (17.5 µg/kg) resembled those obtained with a model-suggested periodic protocol of a much smaller dose (2 µg/kg on 4 consecutive days). In rhesus monkeys, treated by rhesus monkey recombinant TPO (5 µg/kg on 4 consecutive days), the suggested protocol yielded effective platelet stimulation with minimal immunogenicity, as demonstrated over prolonged application (24,25)

### Oncology personalization

A prevailing notion in oncology is that the progression of cancer in a given individual is difficult if not impossible to predict. Indeed, the manifestations of disease vary substantially – seemingly haphazardly – across different patients, owing to factors such as age, gender, diet, organ function, and genetic variability. It follows that a patient's response to a given therapeutic regimen is also virtually impossible to predict (26,27). Current paradigms of clinical trials do little to address this issue: new drug candidates are administered through exactly the same protocol (or through a limited number of protocols) to large populations of patients and are approved for further consideration if they yield statistically significant improvement, that is, enough patients are benefitted. As a result, drug candidates that might suit small fractions of the patient population, or that might yield benefit to certain patients if administered in a specific protocol that differs from protocols tested slip through the cracks.

Although cancer and response to cancer therapy are indeed highly individual, they are not necessarily unpredictable. Our working hypothesis is that the important pathological processes are relatively consistent across patients and can be described by the same formulas. But, although the general formulas are similar in all patients, the values of the parameters in these formulas, that is, the various reaction rates are patient-specific. The different reaction rates, typifying the pathophysiological processes in each patient, are the source of variation in the response to treatment. As discussed above, in order to design an effective drug therapy approach for an individual patient – it is important to know the patient-specific reaction rates of the influential pathological and physiological processes (eg, cell cycle duration when chemotherapy is involved).

Once certain clinical data have been collected, mathematical models can be used to estimate the patient's individual parameter values and to identify the optimal treatment regimen for him or her. Such models can take into account not only the patient's characteristics but also the specific aim of the treatment: for example, in some cases the clinician may seek to aggressively eliminate the tumor, even at the risk of side effects, whereas in others it may be sufficient merely to slow down tumor progression while prioritizing avoidance of toxicity. The use of mathematical models can transform the process of identifying an effective treatment regimen from one of trial and error into a rational decision-making process (10,18).

A case study by Gorelik et al (12) demonstrates the crucial role of personalization in the design of effective resonance-based dosing regimens. The researchers carried out their study in a single patient, a 45-year-old white man suffering from mesenchymal chondrosarcoma (MCS). MCS is a rare disease (accounting for about 1% of chondrosarcomas) with a 5-year survival rate of 55%, for which there is currently no efficacious treatment. The patient had undergone aggressive chemotherapy with ifosphamide, cisplatin, and etoposide for six cycles, VACA (vincristine, doxorubicin, cyclophosphamide, and dactinomycin) for 2 cycles, and sunitinib p.o. for 8 weeks. Despite this treatment regimen, the cancer continued to spread, with additional metastases appearing in the liver and bone. Furthermore, as a result of the aggressive treatment, the patient suffered severe myelo-suppression with pancytopenia.

**Figure 4:**
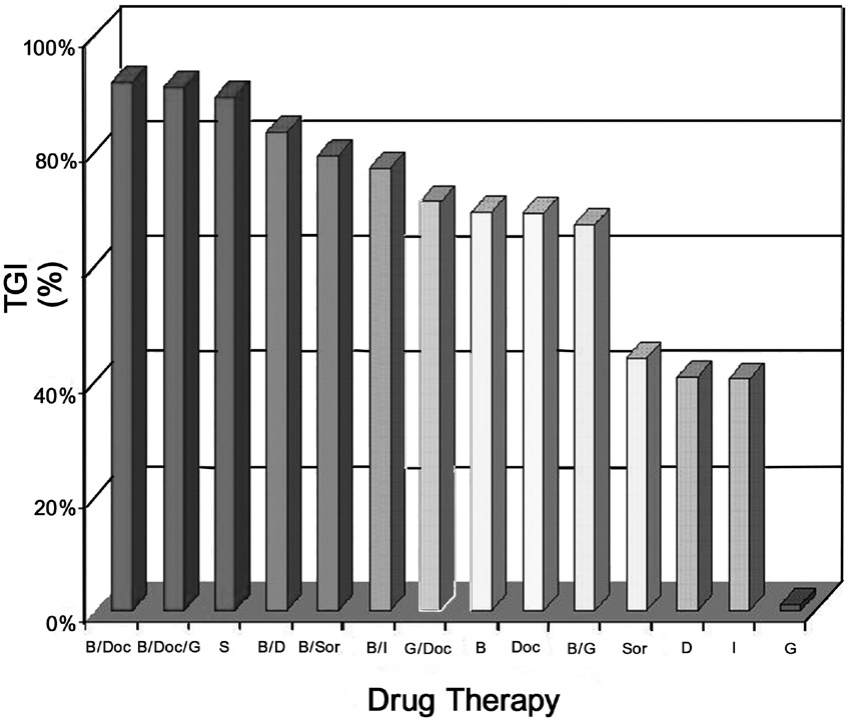
Mathematical model predictions of tumor growth inhibition (TGI). The drug protocols that were simulated are shown at the bottom of each histogram bar: B/Doc denotes bevacizumab, 10 mg/kg, IV, Q3Dx10 +docetaxel, 25 mg/kg, IV, Q7Dx3; B/Doc/G denotes bevacizumab, 6.7 mg/kg, IV, day 1,8 + docetaxel, 25 mg/kg, IV, Q7Dx3 + gemcitabine, 160 mg/kg, IV infusion, 24 hours (single dose); S denotes sunitinib, 40 mg/kg, PO b.i.d x28; B/D denotes bevacizumab, 5 mg/kg, IP, Q4Dx6 + docetaxel, 3 mg/kg, IV, QDx8; B/Sor denotes bevacizumab, 5 mg/kg, IP, Q4Dx6 + Sorafenib, 85 mg/kg, PO, QDx10.

To collect clinical data regarding the progression of the cancer, the researchers xenografted tumor samples, obtained from the patient's lung, into mice. They measured tumor volume over time and analyzed gene expression in the xenografted tumors as well as in the patient's metastases. Once tumors grew to 50-150 mm^3^ in size, animals were pair-matched by tumor size into treatment and control groups, and treatment animals were administered drugs by different monotherapy or combination regimens. In parallel, a general growth model of vascular tumors was adjusted to describe the CS xenograft dynamics. This was done by evaluating the parameters of tumor growth dynamics in the untreated mice. Using the xenograft-adjusted model, the growth of the MCS xenografts and their response to various drug therapies was simulated, in conjunction with the pharmacokinetics and the pharmacodynamics of the pertinent drugs, and with the applied dosing regimens. The developed model was used to evaluate tumor growth inhibition (TGI) in the xenografted MCS patient tumors, by simulating different monotherapies and combinations of two or three cytotoxic and anti-angiogenic drugs (TGI is a conventionally-formulated measurement of the change, over time, in tumor size under a particular drug protocol, as compared to the change in untreated tumors over the same time). Model simulations enabled predictions of the differences in TGI which would be inflicted by the different drug protocols, applied to the MCS patient’s xenografts. The combination of bevacizumab and docetaxel was predicted to be most efficacious in inhibiting the growth of tumors originating at the MCS lung metastasis (Figure 4). Model predictions were compared to the experimentally observed values, showing prediction accuracy of 87.1%.

The personalized MCS xenograft model was the basis for the human model. Model parameters were up-scaled by comparing experimentally evaluated gene expression levels of several growth factors in the patient to those in mice and adjusting parameter values accordingly. Literature data on the involved drugs were employed to model their pharmacokinetics in this MCS patient. Thereafter, a mathematical MCS patient model was simulated to predict the patient’s response to many different docetaxel/bevacizumab combination regimens. Simulation results suggested that a regimen containing bevacizumab applied i.v. in combination with once-weekly docetaxel would be more efficacious in the MCS patient than any other simulated schedule. Indeed, weekly docetaxel in the patient stabilized the metastatic disease and mitigated pancytopenia. This result was not surprising. The knowledge that the major processes involved in solid tumor progression have periodic patterns, in conjunction with the universality of the resonance phenomenon, imply that there exists an optimal docetaxel regimen of a characteristic period. Docetaxel is a microtubule stabilizing drug. In addition to its cytotoxicity against tumor cells, it also exhibits antiangiogenic properties when used at low doses, by inhibiting endothelial cell proliferation, migration, and tube formation (28). Gorelik et al conjectured that angiogenesis was the force that drove the efficacy of docetaxel.

In order to apply the above theoretical results in treatment personalization, it is important to verify that, indeed, the superiority of the once-weekly docetaxel, as compared to the standard tri-weekly regimen, is determined by the clinical angiogenic parameters of the individual patient. To this end, Gorelik et al (12) formulated an arbitrary function of the kinetic rate of new vessel formation, as influenced by vascular endothelial growth factor (VEGF) secretion, density of VEGF receptors, etc (denoted angiogenesis scaling factor and representing the intensity of angiogenesis). By simulating in the mathematical MCS model virtual patients carrying different values of the Angiogenesis Scaling Factor, they could test the potential effect of angiogenesis on the efficacy of docetaxel with or without bevacizumab, as measured by TGI effect over time. Doing so, they could show that superiority of the one-weekly docetaxel regimen, over the tri-weekly regimen is a non-monotonic function of the angiogenic scaling factor (Figure 3B in[12]). This result, demonstrating the resonance phenomenon in the effects of a drug on the progression of a vascular tumor, shows the importance of the dosing period in the efficacy of a treatment regimen and provides further support to the existence of the resonance phenomenon in human cancer.

In general, the case study by Gorelik et al (12) points to the enormous potential of personalization in the design of treatment regimens that take resonance into account, particularly in the context of rare diseases such as MCS, for which current clinical trial paradigms are particularly ill-suited. As yet, while personalized medicine is gradually gaining recognition, individual clinicians still do not exploit anywhere near its full potential in practice.

## Conclusion

Mathematical modeling has revealed the resonance phenomenon in population persistence, whereby small external forces applied to an oscillating biological system can produce large effects on the size of the population – provided that the period of these forces is synchronized with the period of the population. First identified in the context of intertidal mollusks in the Red Sea, the resonance phenomenon has been shown to characterize seemingly dissimilar phenomena such as the spread of pathogenic disease in a population as well as cancer progression, or hematopoiesis. In epidemiology, policy makers can achieve cost savings and substantially improve efficacy by adopting a “pulse” vaccination policy, which is vaccinating small fractions of the population at specific intervals, instead of adopting a cohort-based policy or a one-time mass vaccination policy. In the case of cancer, a resonance-based chemotherapeutic approach, designed according to the patient's individual characteristics, can substantially limit tumor progression while simultaneously avoiding damage to healthy cells. This theory holds tremendous practical implications as well as broader significance: it shows that, fundamentally, biological domains that seem completely unrelated to one another are actually very similar.
